# Regulation of Smad2/3 Nuclear Exclusion by Follicle-Stimulating Hormone (FSH) in Chicken Follicular Granulosa Cells and Its Effect on FOXO3/4

**DOI:** 10.3390/genes16030283

**Published:** 2025-02-26

**Authors:** Yuhan Sun, Simushi Liswaniso, Hengsong Wu, Xue Sun, Chunchi Yan, Ning Qin, Rifu Xu

**Affiliations:** 1 Joint International Research Laboratory of Modern Agricultural Technology, Ministry of Education, Jilin Agricultural University, Changchun 130118, China; sunyh990406@163.com (Y.S.); smliswaniso@gmail.com (S.L.); wx15298288966@163.com (H.W.); xuesun1128@163.com (X.S.); 18302478816@163.com (C.Y.); qinninglove2008@126.com (N.Q.); 2 Department of Animal Genetics, Breeding and Reproduction, College of Animal Science and Technology, Jilin Agricultural University, Changchun 130118, China

**Keywords:** chicken, follicular granulosa cells, FSH, FOXO3/4, Smad2/3, nuclear exclusion

## Abstract

Background: This study aims to investigate the regulation of small mothers against decapentaplegic 2 and 3 (Smad2/3) protein phosphorylation and nuclear exclusion in follicular granulosa cells (GCs) by chicken follicle-stimulating hormone (FSH) through the phosphatidylinositol 3-kinase (PI3K) signaling pathway, as well as the effect of Smad2/3 proteins on forkhead box O 3 and 4 (FoxO3/4). This lays the foundation for exploring the regulatory functions of signaling pathways closely related to follicular growth and development, as well as the molecular mechanisms of subcellular localization and nuclear exclusion of various effector factors (including transcription factors). Methods: In this study, we used granulosa cells from 6–8 mm prehierachical follicles of chickens and performed immunofluorescence, quantitative real-time PCR (RT-qPCR), and Western blotting analysis to detect the phosphorylation and nuclear exclusion of Smad2/3 induced by FSH, as well as the regulatory effect of Smad2/3 on FOXO3/4 proteins. Results: The results showed that 10 ng/mL FSH and 50 μg/mL PI3K activator significantly reduced the phosphorylation level of Smad2/3 (*p* < 0.05), while no nuclear exclusion was observed. On the other hand, 16 nM/mL PI3K inhibitor and 50 μg/mL alkaline phosphatase significantly increased the phosphorylation level of Smad2/3 (*p* < 0.05). Overexpression of Smad2/3 increased the phosphorylation level of FOXO3/4 (*p* < 0.05); Smad2/3 interference resulted in a decrease in FOXO3/4 phosphorylation levels (*p* < 0.05). Conclusions: FSH can inhibit Smad2/3 phosphorylation and retain it in the nucleus through the PI3K signaling pathway. Smad2/3 and FOXO3/4 act as downstream effectors of the PI3K signaling pathway, and Smad2/3 can promote the phosphorylation of FOXO3/4.

## 1. Introduction

Follicle-stimulating hormone (FSH) is a glycoprotein synthesized and secreted by the anterior pituitary gland, which binds to the receptors on granulosa cells (GCs) and can promote gonadal development in livestock and poultry by facilitating hormone synthesis in granulosa cells [1,2]. Forkhead box O 3 and 4 (FoxO3/4) are members of the forkhead box O transcription factor family (FOXO), which mainly functions to inhibit the cell cycle [3]. They can integrate signals from multiple signaling pathways to regulate cell differentiation, cell cycle progression, and programmed cell death. Phosphorylated FOXO3/4 proteins can inhibit the transcription function of pro-apoptotic genes and promote granulosa cell proliferation [4]. Small mothers against decapentaplegic 2 and 3 (Smad2/3) proteins, as members of the Smads protein family receptor regulation, are primarily involved in signal transduction within the activator signaling pathway, closely regulating cell cycle progression and apoptosis induction. Smad2/3 plays an important role in embryonic development, participating in the regulation of ovulation occurrence and development; it also plays an important role in the transition from primordial follicles to primary follicles and the formation of antral follicles [5,6,7]. Smad2/3-dependent transcription can be affected by FOXOs [8]. On the contrary, phosphorylated Smads activate FOXOs through dephosphorylation [9]. As Smads partner in cell cycle regulation, FOXOs act as signal transducers at the confluence of Smads and phosphatidylinositol 3-kinase (PI3K) pathways, thus integrating the Smads and PI3K signaling pathways [10,11]. In mammals, the interaction between FOXOs and Smads helps to control the proliferation of neuroepithelial cells and glioblastoma cells, and the inhibition of PI3K signaling leads to reduced cell proliferation [10]. Smads interact with FOXOs and activate them to inhibit cell apoptosis.

Nuclear exclusion refers to the removal or retention of certain phosphorylated protein molecules (such as certain transcriptional activation/inhibition factors) from the nucleus or cytoplasm, respectively, under the action of specific protein kinases, thus preventing their binding to their target gene’s cis-acting elements and exerting transcriptional activation or inhibition [12,13]. However, there are no reports on the mechanism of FSH regulation of the Smad2/3 factor and its impact on FOXOs in chicken follicular granulosa cells. Therefore, this study investigated the regulatory effect of FSH on Smad2/3 protein phosphorylation and nuclear exclusion in chicken follicular granulosa cells through the PI3K signaling pathway, as well as the effect of Smad2/3 on FOXO3/4. The study explores the molecular mechanism of the nuclear exclusion of transcription factors closely related to follicular growth and development, which could enhance our biological comprehension of these complex processes.

## 2. Materials and Methods

### 2.1. Ethics Statement

All procedures involving chickens in the extant study were authorized by the Institutional Animal Care and Use Committee (IACUC) at Jilin Agricultural University (Changchun, China). The experiments adhered to the ARRIVE guidelines [14]. Prior to organ removal, chickens were euthanized through decapitation following the IACUC’s protocols for experimental animals (Permission No. GR (J) 19-030). The euthanasia process fully complied with relevant Chinese legislation and regulations on laboratory animal care and use, as stipulated in the 2017 revision of the Regulations for the Administration of Experimental Animal Affairs by the State Council of the People’s Republic of China.

### 2.2. Study Design, Current Sampling, and Granulosa Cell Culture

The experiment employed a completely randomized design, in which experimental hens of the same age, breed, and feeding regimen were randomly assigned to either the treatment group or the control group. For this study, twenty Lohman Brown laying hens aged 21 weeks raised at Jilin Agricultural University Provincial and Ministerial Key Laboratory Experimental Chicken Farm (Changchun, China) were used. All hens were raised similarly according to previously reported standards [15]. After the hens’ acquisition, they were euthanized, and follicles with a diameter between 6 and 8 mm were collected and stored in sterile containers.

Follicular granulosa cells (GCs) were then isolated from the follicles according to published methods [16]. The GCs were then incubated at a concentration of 1 × 10^6^/mL at 37 °C, 5% CO_2_, and relative saturation humidity of 95%. The microscope was directly used to observe the morphology and quantity of the cells and to evaluate the survival status of cells based on their shape. The cells were cultured with 10 ng/mL FSH and 50 μg/mL PI3K activator (Selleck Chemicals, Houston, TX, USA) for 12 h in the presence or absence of 16 nM/mL PI3K inhibitor (Med Chem Express, Monmouth Junction, NJ, USA) and 50 μg/mL alkaline phosphatase (Yuanye, Beijing, China) [17,18]. The concentration of reagents was determined according to the instructions or references provided in the user manual.

The cells in the control group received no treatment. Using the described cell culture method, the four distinct drug stimulation protocols were assigned to four separate treatment groups. A total of 20 hens were used, from which 26 cell culture plates were prepared, with 6 wells per plate and 1 replicate per well. For RT-qPCR, 5 duplicate samples were set for each condition, and 3 duplicate samples were used for Western blot (WB) and immunofluorescence (IF).

### 2.3. Immunofluorescence Staining

In this study, circular glass slides were placed in six-well cell culture plates to facilitate cell growth onto the slides. The cells were fixed with 4% paraformaldehyde (Beyotime, Shanghai, China), and the edges of the glass slides were uniformly coated with an immunohistochemical pen to create a 1 mm oily edge. Primary antibodies against Smad2 (Bioss, Wuhan, China, 1:100 dilution) and Smad3 (Bioss, Beijing, China, 1:100 dilution) were applied to the cells, and the cells were incubated at 4 °C for 12 h. Following phosphate buffer saline washes, the cells were treated with a fluorescently conjugated goat anti-rabbit IgG antibody (Epizyme Biotech, Shanghai, China, 1:1000 dilution). The cell nuclei were stained with 4′,6-diamidino-2-phenylindole (DAPI, Epizyme Biotech, Shanghai, China), and immunofluorescence images were captured using a confocal laser microscope (Zeiss, Oberkochen, Germany).

### 2.4. Western Blot Analysis

The cells were treated with RIPA cell lysate (Biosharp Life Sciences, Hefei, China) to extract protein samples. Protease inhibitors (Epizyme Biotech, Shanghai, China) and phosphatase inhibitors (Epizyme Biotechnology, Shanghai, China) were subsequently added, and ultrasonic fragmentation was performed. The protein concentration was adjusted using a BCA assay kit (Beyotime, Shanghai, China), following the manufacturer’s instructions. Protein samples were separated using 10% SDS-PAGE gel (Epizyme Biotech, Shanghai, China), and the proteins were transferred to membranes after electrophoresis at 150 V.

The membranes were blocked with 5% skim milk powder and incubated overnight at 4 °C with the following primary antibodies: anti-FOXO3 (Proteintech, Wuhan, China, 1:2000 dilution), p-FOXO3 (Bioss, Beijing, China, 1:1000 dilution), anti-FOXO4 (Bioss, Beijing, China, 1:1000 dilution), p-FOXO4 (Bioss, Beijing, China, 1:1000 dilution), anti-Smad2 (Bioss, Beijing, China, 1:1000 dilution), p-Smad2 (Bioss, Beijing, China, 1:1000 dilution), anti-Smad3 (Bioss, Beijing, China, 1:1000 dilution), p-Smad3 (Bioss, Beijing, China, 1:1000 dilution), and anti-β-actin (Boster Biological Technology, Wuhan, China, 1:10,000 dilution). After washing with PBS, the membranes were incubated for 1.5 h with goat anti-mouse IgG conjugated with horseradish peroxidase as the secondary antibody. Protein bands were visualized using the ECL Plus Western blot detection system.

### 2.5. Transfection of Overexpression and Interference Vectors

The Smad2/3 overexpression vector and interference vector used in this experiment were designed by GenePharma Biotech (Shanghai, China). Plasmid transfection of granulosa cells was performed according to standard methods of Lip2000 (Biosharp Life Sciences, Heifei, China).

### 2.6. Quantitative Real-Time RT-PCR

To verify if the overexpression vector and interference vector were successfully transfected into cells, real-time quantitative reverse transcriptase PCR (qRT-PCR) was performed according to the methods reported by Xu et al. [14]. The primer sequences used in this study are shown in Table 1. The reaction system contained 10 μL of SYBR, 0.4 μL of upper and lower primers, and 2 μL of cDNA. The amplification conditions used were 95 °C for 10 min and 95 °C for 15 s, with an annealing temperature of 60 °C for 60 s, for a total of 40 cycles. The 18S rRNA gene was used as an internal control to normalize the expression levels of the target genes using the 2^−ΔΔCt^ method [19]. The same sample includes 18S rRNA and the target gene in the same batch of reactions, with 5 replicates for each reaction, and each sample was tested twice. The relative expression level of mRNA was calculated according to the following standard formula: 2^−ΔΔCt^ = 2[−(ΔCt sample − ΔCt control)].

### 2.7. Statistical Analysis

All data were replicated twice and analyzed using SPSS 12.0 [15]. The dependent variables measured in this experiment included relative mRNA expression levels, cellular fluorescence localization, and relative protein expression levels. A student *t*-test was used to assess the difference between the treatments and the control. Significant differences between treatments were considered when *p* < 0.05.

## 3. Results

### 3.1. FSH Induces Smad2/3 Phosphorylation and Nuclear Exclusion Through the PI3K Signaling Pathway

**Figure 1:**
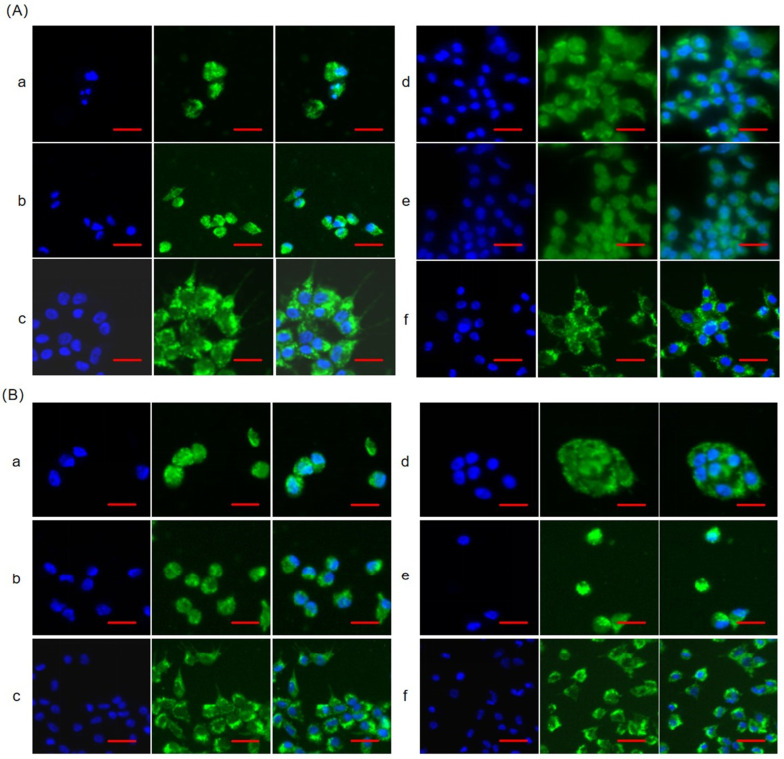
The nuclear exclusion of Smad2/3 in the cultured granulosa cells (GCs) of the ovarian follicles. (**A**) Immunofluorescence detection of Smad2 subcellular localization in the GCs. Smad2 is shown in green, and nucleus is stained with 4′,6-diamidino-2-phenylindole (DAPI) (blue). (**B**) Immunofluorescence detection of Smad3 subcellular localization in the GCs. Smad3 is shown in green, and nucleus is stained with DAPI (blue). a. control, without any treatment; b. treatment of the cells with 10 ng/mL follicle-stimulating hormone (FSH) for 12 h; c. co-treatment of the cells with 10 ng/mL FSH and phosphatidylinositol 3-kinase (PI3K) inhibitor for 12 h. d. control, without any treatment; e. treatment of the cells with 50 μg/mL PI3K activator for 12 h; f. co-treatment of the cells with 50 μg/mL PI3K activator and 50 μg/mL alkaline phosphatase for 12 h. *n*  =  3 independent experiments. Leica DMI8, 400×; scale bar  =  5 μm.

To reveal the effect of FSH signaling through the PI3K pathway on Smad2/3 protein phosphorylation and nuclear exclusion in chicken ovarian GCs, Western blot was used to detect and analyze the levels of phosphorylated Smad2/3 (p-Smad2/3), and their subcellular localization in cultured GCs was simultaneously examined by immunofluorescence analysis. As shown in Figure 1, after the treatment of the cells with 10 ng/mL FSH, Smad2/3 showed no significant nuclear exclusion, but was distributed both inside and outside the cell nucleus. After treating the cells with 10 ng/mL FSH + 16 nM/mL PI3K inhibitor, the nuclear fluorescence intensity significantly decreased, and Smad2/3 underwent nuclear exclusion. After the treatment of the cells with 50 μg/mL PI3K activator, Smad2/3 did not exhibit significant nuclear exclusion. After treating the cells with 50 μg/mL PI3K activator and 50 μg/mL alkaline phosphatase, the fluorescence intensity inside the nucleus decreased, and Smad2/3 underwent nuclear exclusion.

**Figure 2:**
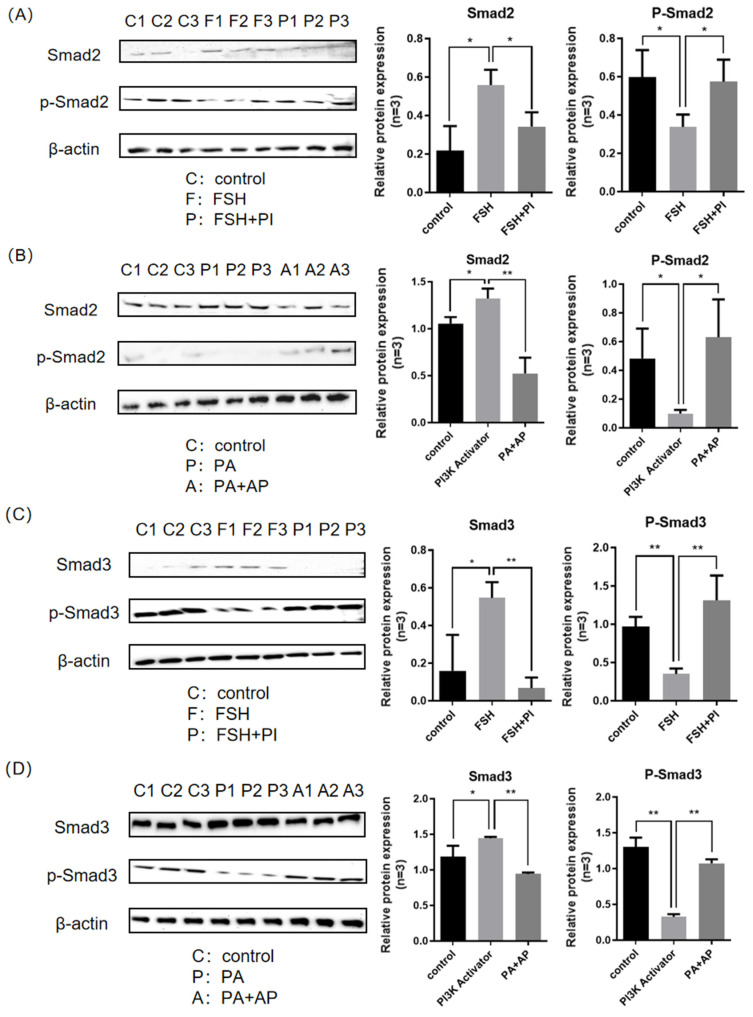
Smad2/3 phosphorylation in the cultured GCs of the ovarian follicles. (**A**) The Smad2 and p-Smad2 levels were determined by Western blotting analysis in the GCs without any treatment, treated with 10 ng/mL FSH for 12 h, and co-treated with 10 ng/mL FSH and 16 nM/mL PI3K inhibitor for 12 h. (**B**) The Smad3 and p-Smad3 levels were tested in the GCs without any treatment, treated with 10 ng/mL FSH for 12 h, and co-treated with 10 ng/mL FSH and 16 nM/mL PI3K inhibitor for 12 h. (**C**) The Smad2 and p-Smad2 levels were determined by Western blotting analysis in the GCs without any treatment, treated with 50 μg/mL PI3K activator for 12 h, and co-treated with 50 μg/mL PI3K activator and 50 μg/mL alkaline phosphatase for 12 h. (**D**) The Smad3 and p-Smad3 levels were tested in the GCs under the following conditions: without any treatment, treated with 50 μg/mL PI3K activator for 12 h, and co-treated with 50 μg/mL PI3K activator and 50 μg/mL alkaline phosphatase for 12 h. The data represent the mean ± SEM (*n* = 3). Student’s *t*-test, ** *p*  <  0.01 and * *p*  <  0.05.

As shown in Figure 2A,C, after treatment with 10 ng/mL FSH, the expression level of Smad2/3 proteins significantly increased (*p* < 0.05), while the expression level of p-Smad2/3 proteins significantly decreased (*p* < 0.05). After treatment with 10 ng/mL FSH + 16 nM/mL PI3K inhibitor, the expression level of Smad2/3 proteins was significantly reduced (*p* < 0.05), and the expression level of phosphorylated proteins was significantly increased (*p* < 0.05). As shown in Figure 2B,D, after treatment with 50 μg/mL PI3K activator, the expression level of Smad2/3 proteins significantly increased (*p* < 0.05), while the expression level of p-Smad2/3 proteins significantly decreased (*p* < 0.05). After treatment with 50 μg/mL PI3K activator and 50 μg/mL alkaline phosphatase, the expression level of Smad2/3 proteins was significantly reduced (*p* < 0.05), while the expression level of phosphorylated protein was significantly increased (*p* < 0.05). The above results indicate that FSH inhibits the phosphorylation and nuclear exclusion of Smad2/3, and this process is regulated through the PI3K signaling pathway.

### 3.2. The Regulatory Effect of Smad2/3 on FOXO3/4 Protein Phosphorylation

**Figure 3:**
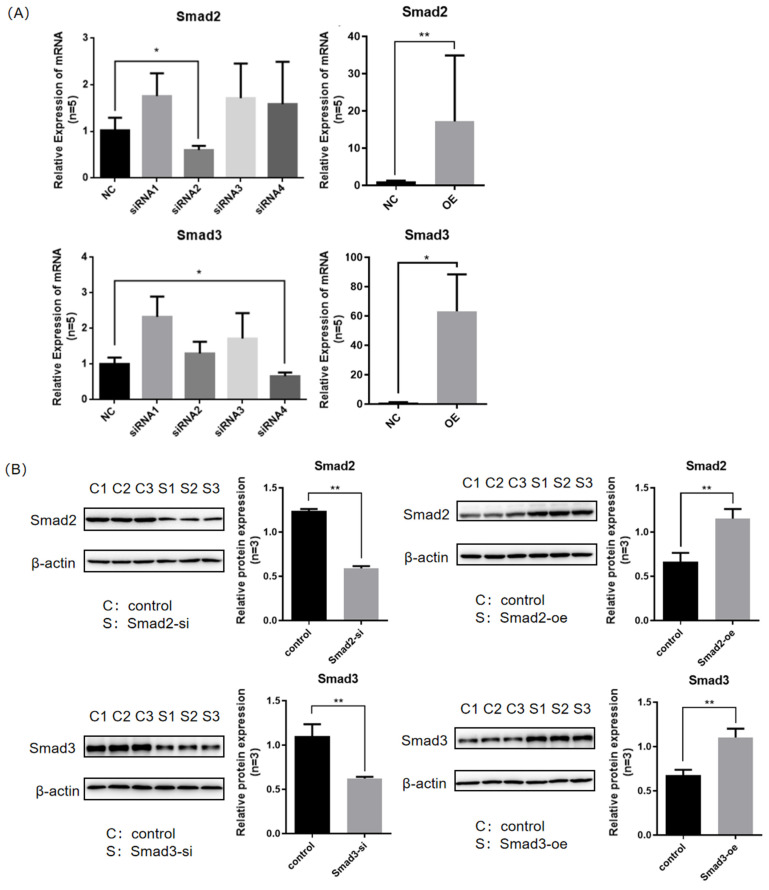
Detection of Smad2/3 interference and overexpression vector transfection effect. (**A**) The mRNA levels of Smad2/3 were determined by qRT-PCR in GCs under interference and overexpression plasmid transfection and non-transfection conditions. The data represent mean ± SEM (*n* = 5). Student’s *t*-test, ** *p*  <  0.01 and * *p*  <  0.05. (**B**) The protein levels of Smad2/3 were determined by Western blotting analysis in GCs under interference and overexpression plasmid transfection and non-transfection conditions. The data represent mean ± SEM (*n* = 3). Student’s *t*-test, ** *p*  <  0.01 and * *p*  <  0.05.

To investigate the regulatory effect of Smad2/3 on FOXO3/4 proteins, interference and overexpression treatments were performed on the Smad2/3 gene. Firstly, qRT-PCR and Western blot were used to detect the mRNA and protein expression levels of the target gene to verify whether the Smad2/3 overexpression vector and interference vector were successfully transfected into the cells. Through PCR experiments (Figure 3A), it was found that the mRNA expression level in the Smad2 siRNA2 transfection group was significantly reduced (*p* < 0.05), with an interference efficiency of 58.9%. The mRNA expression level in the Smad3 siRNA4 transfection group was significantly reduced (*p* < 0.05), with an interference efficiency of 65.7%. The expression level of Smad2 mRNA in the group transfected with the Smad2 overexpression vector was significantly increased (*p* < 0.05), with an overexpression efficiency of 31.9 times. The expression level of Smad3 mRNA in the group transfected with the Smad3 overexpression vector was significantly increased (*p* < 0.05), and the overexpression efficiency was 62.1 times. The respective protein expression in the four groups of samples was detected, and Western blot experiments (Figure 3B) showed that the Smad2/3 protein expression levels in the interference vector and overexpression vector transfection groups were significantly changed (*p* < 0.05). This result indicates that the Smad2/3 overexpression vector and interference vector were successfully transfected, and the samples could be used for subsequent experiments.

**Figure 4:**
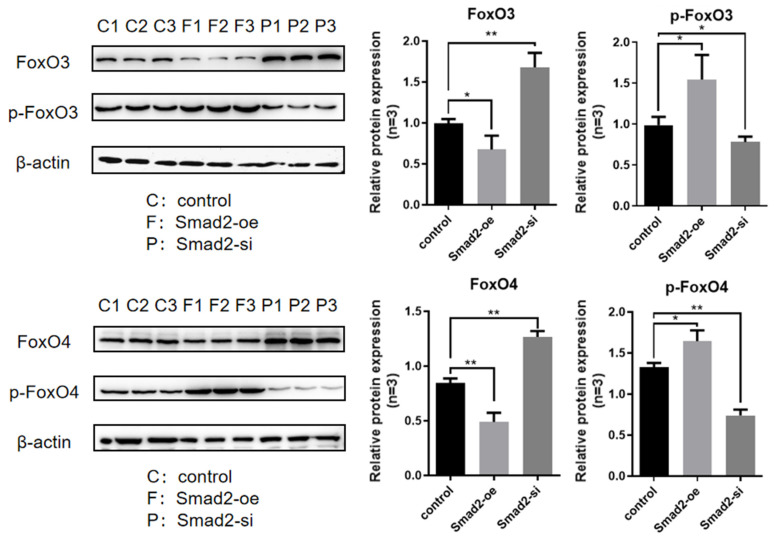
Effects of Smad2 on the forkhead box O 3 and 4 (FoxO3/4) phosphorylation in the cultured GCs of the ovarian follicles. The FOXO3/4 and p-FOXO3/4 levels were determined by Western blotting analysis in the GCs with Smad2 overexpression and interference. The two treatment groups were compared with the control group separately, but there was no comparative analysis between the treatment groups. The data represent mean ± SEM (*n* = 3). Student’s *t*-test, ** *p*  <  0.01 and * *p * <  0.05.

**Figure 5:**
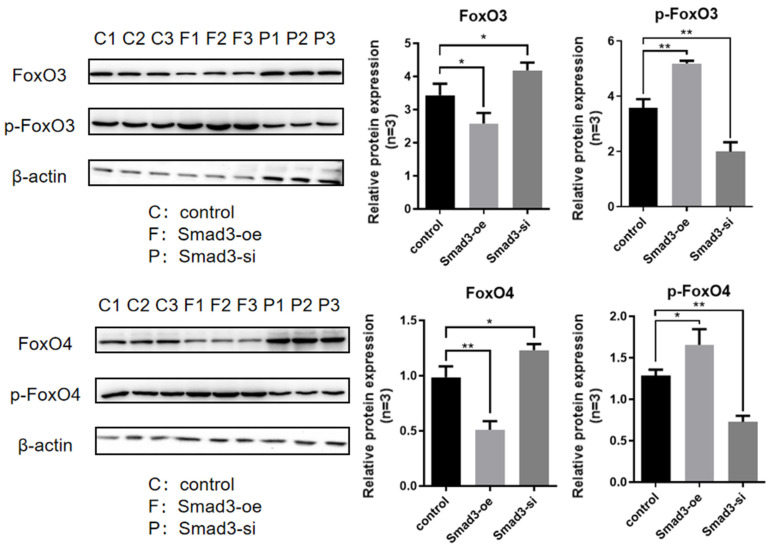
Effects of Smad3 on the FOXO3/4 phosphorylation in the cultured GCs of the ovarian follicles. The FOXO4 and p-FOXO4 levels were determined by Western blotting analysis in the GCs with Smad3 overexpression and interference. The two treatment groups were compared with the control group separately, but there was no comparative analysis between the treatment groups. The data represent mean ± SEM (*n* = 3). Student’s *t*-test, ** *p*  <  0.01 and * *p*  <  0.05.

Through experimental observation (Figure 4), it was found that the overexpression of Smad2 significantly reduced the expression level of FOXO3/4 proteins (*p* < 0.05), while the expression level of p-FOXO3/4 proteins significantly increased (*p* < 0.05). After interfering with Smad2, the expression level of FOXO3/4 proteins significantly increased (*p* < 0.05), while the expression level of p-FOXO3/4 proteins significantly decreased (*p* < 0.05). Through experimental observation (Figure 5), it was found that the overexpression of Smad3 significantly reduced the expression level of FOXO3/4 proteins (*p* < 0.05), while the expression level of p-FOXO3/4 proteins significantly increased (*p* < 0.05). After interfering with Smad3, the expression level of FOXO3/4 proteins significantly increased (*p* < 0.05), while the expression level of p-FOXO3/4 proteins significantly decreased (*p* < 0.05). This phenomenon indicated that an increase in Smad2/3 protein expression promotes the phosphorylation of FOXO3/4; after a decrease in Smad2/3 protein expression, the phosphorylation of FOXO3/4 proteins was inhibited. The above results indicate that Smad2/3 proteins have a regulatory effect on the expression and phosphorylation of FOXO3/4 proteins. It negatively regulates the expression of FOXO3/4 proteins, promotes their phosphorylation, and, thus, inhibits their transcription factor activity.

## 4. Discussion

The egg production performance of chickens largely depends on the gradual stage of follicle growth and development [20], with the role of 6–8 mm prehierarchical follicles being the most important [21]. Granulosa cells have a significant impact on the development of follicles. FOXO3/4 and Smad2/3 regulate downstream signaling factors in the nucleus, thereby affecting the physiological processes of the granulosa cells [22], while the phosphorylation and nuclear exclusion of FOXO3/4 and Smad2/3 affect the progression of this process. The body regulates the PI3K signaling pathway through FSH; FOXO3/4 and Smad2/3, as downstream signaling molecules of this pathway, are also regulated by it. Smad2/3 also have an effect on the FOXO3/4 proteins. However, this phenomenon has not been studied in the granulosa cells of chicken follicles. This study indicates that in chicken granulosa cells, FSH regulates the PI3K signaling pathway to promote the FOXO3/4 nuclear exclusion but inhibits Smad2/3 protein phosphorylation and nuclear exclusion. Smad2/3 proteins inhibit the expression of FOXO3/4 proteins, promoting their phosphorylation.

FSH regulates the PI3K signaling pathway to inhibit the Smad2/3 phosphorylation and nuclear exclusion. At present, research has shown that PI3K inhibitors can block the phosphorylation of Smad2 in breast cancer cells, which indicates that Smad2 protein is the downstream target of the PI3K pathway [23]. In human mesangial cells, the PI3K pathway actively regulates the transcriptional activity of Smad3, which is phosphorylated by this pathway at the target site of serine residues [24]. Akt, as an important cell survival stimulatory factor, inhibits the Smad3-induced cell apoptosis by interacting with unphosphorylated Smad3 [25,26]. After the phosphorylation of Akt in mouse myoblasts, the phosphorylation level of Smad3 decreases accordingly [27]. In studies related to the expression and localization of Smad2/3 and phosphorylated Smad2/3 in human kidneys, it was found that non-phosphorylated Smad2/3 proteins are mainly distributed in the cytoplasm of renal tubular and collecting duct epithelial cells, while p-Smad2/3 is mainly distributed in the nucleus of the renal cortex and renal module [11]. This indicates a correlation between Smad2/3 phosphorylation and its nuclear exclusion phenomenon. Based on the WB and IF results of the Smad2/3 group in this study, it can be concluded that after FSH acts on the chicken follicular granulosa cells, the expression level of Smad2/3 in the nucleus significantly increases, indicating that the nuclear exclusion phenomenon of Smad2/3 disappears after dephosphorylation. This phenomenon is induced by FSH through the PI3K signaling pathway.

Smad2/3 can promote the phosphorylation of FOXO3/4. Previous studies have shown that phosphorylated Smad2/3 activates FOXO3/4 through dephosphorylation [9]. From the WB results in this experiment, it can be seen that after the increase in Smad2/3 protein expression, the expression of FOXO3/4 proteins significantly decreases, and its phosphorylation level significantly increases; after the decrease in Smad2/3 protein expression, the expression of FOXO3/4 proteins significantly increases, and its phosphorylation level significantly decreases. Currently, many related studies have shown that the PI3K pathway can directly phosphorylate FOXOs [28]. In mouse primordial follicular oocytes, the activation of Smads significantly reduces the entry of FOXO3 into the nucleus, indicating that Smads inhibit the apoptosis of primordial follicular oocytes by downregulating the entry of FOXO3 into the nucleus [29]. In human neuroepithelial and glioblastoma cells, higher levels of myostatin and activin lead to a synchronous increase in the phosphorylated Smad2 and non-phosphorylated FOXO3 levels. Previous experiments have shown that the myostatin–activin Smarts cascade activates the FOXOs transcription factor [10]. In mammals, the synergistic interaction between FOXO3/4 and Smad2/3 has been confirmed to regulate the cell cycle regulation and stress response [30].

In these experiments, we focused on the pathway where FSH regulates the phosphorylation and nuclear exclusion of the Smad protein in chicken granulosa cells, namely the PI3K/Akt signaling pathway, and the effect of Smad2/3 on the FoxO3/4 proteins. However, the function and mechanism of action of the FoxO3/4 proteins in follicular granulosa cells are still unknown. Therefore, in subsequent experiments, we will further investigate the effects of FSH on the FoxO3/4 proteins in the granulosa cells, as well as its regulatory effects on granulosa cell proliferation, differentiation, and apoptosis.

## 5. Conclusions

In summary, the results of this study indicate that FSH promotes the expression of the downstream signaling molecules, Smad2/3 proteins, through the PI3K signaling pathway, inhibits their phosphorylation and nuclear exclusion, and causes them to remain in the nucleus. The Smad2/3 and FOXO3/4 proteins jointly act as downstream factors of the PI3K signaling pathway. Smad2/3 can promote the phosphorylation and nuclear exclusion of FOXO3/4 proteins, causing them to lose their function as transcription factors. These findings provide new evidence for FSH’s regulation of Smad2/3 nuclear exclusion and FOXO3/4. This study provides a scientific basis for exploring the molecular regulatory mechanisms related to the growth and development of chicken follicles and has important theoretical significance and application prospects.

## Figures and Tables

**Table 1:** Primer pairs designed for quantitative real-time PCR analysis.

Gene	Forward Primer (5′-3′)	Reverse Primer (5′-3′)
*Smad2*	GTGGTGGAGAACAGAATGGAC	CAGTCCCCAAATTTCAGAGCA
*Smad3*	GAGGAGAAGTGGTGCGAGAAG	GCACTTGGTGTTCACGTTCT
*18s rRNA*	TAGTTGGTGGAGCGATTTGTCT	CGGACATCTAAGGGCATCACA

## Data Availability

The original contributions presented in this study are included in the article. Further inquiries can be directed to the corresponding author.

## Abbreviations

The following abbreviations are used in this manuscript:

FSH	Follicle-stimulating hormone
FSHR	FSH receptor
FOXO	Forkhead box O
PI3K	Phosphatidylinositol-3 kinase
Smad	Small mothers against decapentaplegic
AKT/PKB	Protein kinase B
GC	Granulosa cell
p-FOXO	Phosphorylated FOXO
